# Increased Immunogenicity and Protective Efficacy of Influenza M2e Fused to a Tetramerizing Protein

**DOI:** 10.1371/journal.pone.0046395

**Published:** 2012-10-01

**Authors:** Anne-Marie Carola Andersson, Kjell O. Håkansson, Benjamin Anderschou Holbech Jensen, Dennis Christensen, Peter Andersen, Allan Randrup Thomsen, Jan Pravsgaard Christensen

**Affiliations:** 1 Department of International Health, Immunology and Microbiology, University of Copenhagen, Copenhagen, Denmark; 2 Department of Biology, University of Copenhagen, Copenhagen, Denmark; 3 Department of Infectious Disease Immunology, Statens Serum Institut, Copenhagen, Denmark; Instituto Butantan, Brazil

## Abstract

The ectodomain of the matrix 2 protein (M2e) of influenza A virus represents an attractive target for developing a universal influenza A vaccine, with its sequence being highly conserved amongst human variants of this virus. With the aim of targeting conformational epitopes presumably shared by diverse influenza A viruses, a vaccine (M2e-NSP4) was constructed linking M2e (in its consensus sequence) to the rotavirus fragment NSP4_98–135_; due to its coiled-coil region this fragment is known to form tetramers in aqueous solution and in this manner we hoped to mimick the natural configuration of M2e as presented in membranes. M2e-NSP4 was then evaluated side-by-side with synthetic M2e peptide for its immunogenicity and protective efficacy in a murine influenza challenge model. Here we demonstrate that M2e fused to the tetramerizing protein induces an accelerated, augmented and more broadly reactive antibody response than does M2e peptide as measured in two different assays. Most importantly, vaccination with M2e-NSP4 caused a significant decrease in lung virus load early after challenge with influenza A virus and maintained its efficacy against a lethal challenge even at very low vaccine doses. Based on the results presented in this study M2e-NSP4 merits further investigation as a candidate for or as a component of a universal influenza A vaccine.

## Introduction

Seasonal Influenza A epidemics represent a major threat to the human population worldwide causing three to five million cases of severe illness and about 250,000–500,000 deaths each year (WHO website: http://www.who.int/mediacentre/factsheets/fs211/en/index.html). In addition, there is the constant threat of a pandemic influenza outbreak. Currently available vaccines against seasonal epidemics are efficient as long as there is a good match between the chosen vaccine strains and circulating influenza variants. However, these vaccines target the surface molecule hemagglutinin (HA) and to a lesser extent neuraminidase (NA), and due to the continuous change of these proteins because of mutations and antibody mediated selection, these vaccines need to be updated annually [1]. It is therefore of substantial interest to develop a vaccine that would cross-react between different influenza A virus subtypes and could be used to protect against unpredicted antigenic variation in both epidemic and pandemic outbreaks. Clearly, this type of vaccine should target a conserved viral protein. One promising candidate is the ectodomain of the matrix 2 protein (M2e). The M2 protein is a small protein of 97 amino acids (aa) functioning as a tetrameric ion channel. M2 is involved in uncoating of viral particles in the endosomes and in the maturation of HA in the Golgi apparatus [2]. M2e consists of 23 aa and serves as an attractive target due to the fact that it is highly conserved amongst human influenza A viruses, and with two exceptions no amino-acid change has been found until recently when 4 mutations were identified in the pandemic A California virus [3]–[6]. In addition, M2e-specific antibodies have been found to protect mice, ferrets and non-human primates [7]–[11]. The M2 protein is expressed in low quantities on influenza viral particles, but it is abundantly expressed on the plasma membranes of infected cells [12]. M2e in itself is poorly immunogenic [13] and various strategies have been applied to improve its immunogenicity such as constructing peptide carrier conjugates [8], multiple antigenic peptides (MAPs) [14], [15], M2e-Hepatitis B virus core (M2e-HBc) conjugates [7], recombinant fusion proteins [9], [16], DNA constructs [17], DNA prime-viral boost combinations [18], and delivering combined M2e peptide and split virus [19].

As already mentioned, the natural configuration of M2 is as a tetrameric structure. We were interested in constructing a vaccine based on the native structure of M2 since both monoclonal antibodies [20], [21] against conformational epitopes and a vaccine [16] targeting conformational epitopes of the M2 protein have been proven to afford clinical protection. We therefore replaced the transmembrane part and the cytoplasmic tail of M2 with the NSP4_98–135_ fragment of rotavirus, which due to its coiled-coil region is known to form tetramers in aqueous solution [22]. The fused construct was successfully expressed in *Escherichia coli* (*E. coli)*. For functional analysis, the recombinant fusion protein M2e-NSP4 was compared head-to-head to synthetic M2e peptide, which is the basis for several other M2e targeting vaccines. Here we demonstrate that the vaccine construct M2e-NSP4 is superior to M2e peptide immunization when both are formulated in the liposome based cationic adjuvant CAF-01 [23]–[26], inducing more cross-reactive antibodies and more antibodies that can recognize the native M2 protein. Furthermore, compared to mice immunized with M2e peptide, M2e-NSP4 vaccination significantly reduced both the early viral load in the lungs and the mortality of mice infected with a homologous virus.

## Materials and Methods

### Ethics Statement

Experiments were conducted in accordance with national Danish guidelines (Amendment # 1306 of November 23, 2007) regarding animal experiments as approved by the Danish Animal Experiments Inspectorate, Ministry of Justice, permission numbers 2004/561–867 and 2009/561–1679.

### Recombinant M2e-NSP4 Cloning and Expression

A construct encoding M2e, originating from the human influenza consensus sequence [11], linked to the NSP4_98–135_ fragment of rotavirus, was chemically synthesized with optimized *E. coli* codons (GenScript, NJ). The DNA was amplified from the pUC57 vector (GenScript, NJ) and the polymerase chain reaction (PCR) product was digested with the restriction enzymes NdeI and BamHI (both from Fermentas, Burlington, Ontario) and ligated into a pET11a vector (Novagen, San Diego, CA), which had been digested with the same enzymes. The ligation mixture was electrotransformed into B834(DE3) cells (Novagen, San Diego, CA). Positive clones were confirmed by DNA sequencing (Eurofins, MWG Operon, Germany). The plasmid DNA was then used for retransformation into the same cells and the cultures were grown overnight. The cultures were harvested 3–4 hours after induction with 1 mM IPTG.

### Protein Purification

The bacterial cultures carrying the plasmid pET11a/M2e-NSP4 were grown in LB medium containing 50 µg/mL ampicillin until OD_600_ reached 0.4–0.6. Thereafter, 1 mM IPTG was added for induction for 3–4 hours at 37°C. The cells were harvested by centrifugation at 5000×g for 5 min and resuspended in 50 mM Tris, 50 mM NaCl, pH 7.5. This was followed by centrifugation of the cells at 5000×g for 5 min. The supernatants were removed and the pellets were stored at −20°C.

**Figure 1:**
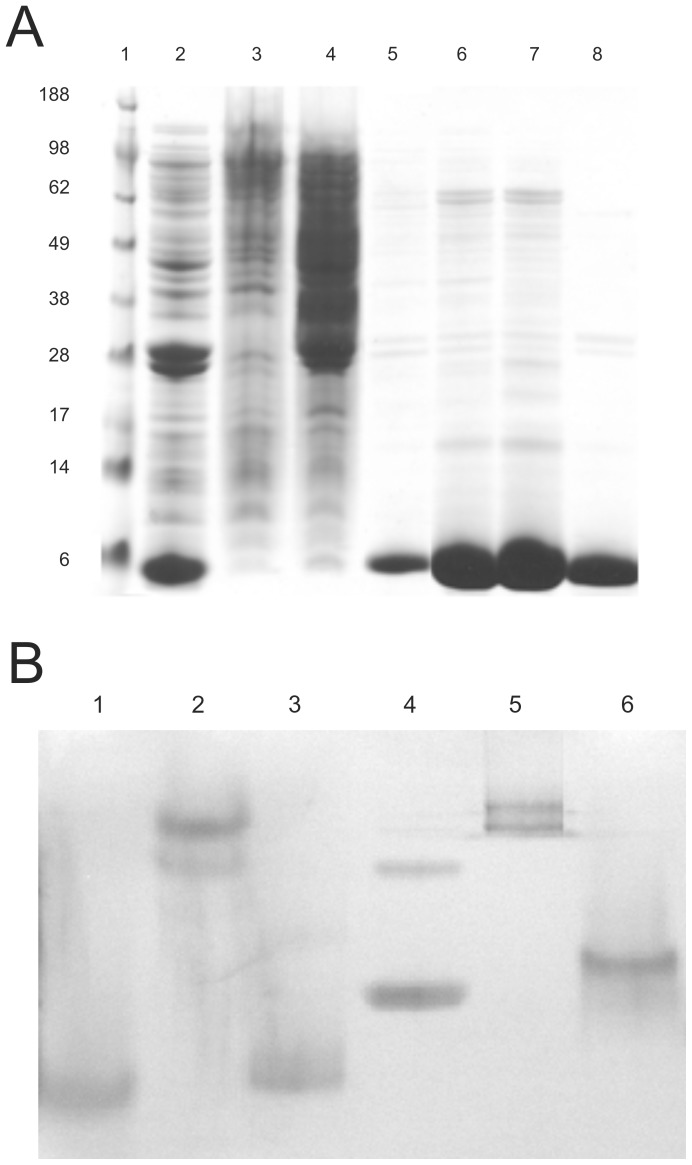
Validation of M2e-NSP4 protein production and purification. A) SDS-PAGE analysis of whole lysate and selected fractions. All samples were run under reduced conditions. Lane 1: molecular weight markers; lane 2: sonicate; lane 3–7: Ni-NTA fractions; lane 8: MonoQ fraction. B) Native PAGE analysis of final pooled fractions. Lane 1: α-Lactalbumin; lane 2: Carbonic Anhydrase; lane 3: Albumin from chicken egg white; lane 4: Albumin from bovine serum; lane 5: Urease; lane 6: M2e-NSP4.

**Figure 2:**
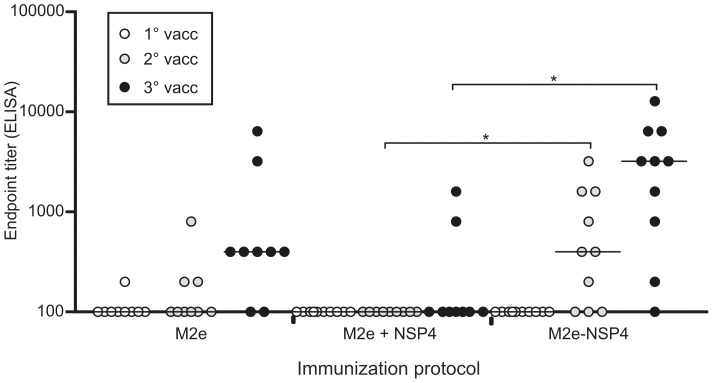
Comparison of the immunogenicity of M2e-NSP4 vs. M2e peptide and an evaluation of the importance of covalent linkage between M2e and NSP4 in the M2e-NSP4 construct. Groups of Balb/c mice (n = 9–10) were immunized at days 0, 21, and 42 with 10 µg of either vaccine formulated in CAF-01. Serum samples were obtained 14 after each vaccination, i.e. at days 14 (1°vacc), 35 (2°vacc) and 56 (3°vacc) after primary vaccination, and analyzed in an ELISA for reactivity against M2e peptide. Results of individual sera are presented; group median titres are indicated by horizontal bars. *denotes a p-value <0.05; the data are representative of at least 5 experiments.

The proteins were purified using nickel affinity chromatography. Specific measures were taken for purification of M2e-NSP4 as this protein only had an HVH-tag positioned at the end of the NSP4 fragment sequence. The bacteria were resuspended in 5 mL of 25 mM Tris, pH 8.0, and sonicated 3×30 s on ice. The suspensions were centrifuged at 18000 rpm × 15 min in the cold. The supernatants were applied to a Tricorn 10/50 column (GE Healthcare, UK), which had been packed with 5 mL nickel-nitrilotriacetic acid (Ni-NTA) (Qiagen). The columns were washed with 1 column volume of 25 mM Tris, pH 8.0, and eluted with 25 mM Tris, 250 mM imidazole, pH 8.0 0–100% over 1 column volume. The relevant fractions were pooled and loaded directly on MonoQ 10/100 columns (Qiagen). The columns were washed with 1 column volume 50 mM Tris, pH 7.5, and the proteins were eluted with 0–1 M NaCl over 5 column volumes. The fractions were stored at −20°C. Analysis on the fractions was done with SDS-PAGE stained with Coommassie Blue A band corresponding to the M2e-NSP4 monomer could be visualized at 7 kDa (Fig. 1). Further analysis of the relevant pooled fractions was done with native PAGE (Fig. 2).

Endotoxin levels were determined using Kinetics Turbidimetric LAL test (Charles River); endotoxin values of the purified protein used in these experiments were ∼4 EU/mg.

### Peptides

The M2e peptides H-MSLLTEVETPTRSEWECRCSDSSDP-OH (A/Ontario/309862/2009(swine-origin H1N1)), H-MSLLTEVETPTRNEWECRCSDSSDP-OH (A/Indonesia/560H/2006 (H5N1)), H-SLLTEVETPIRNEWGSRSNDSSDP-OH (consensus H1N1), H-SLLTEVETPTKSEWESRSNVSSDL-OH (A/equine/London/73 (H7N7)) and the NSP4 fragment H-QMDRVVKEMRRQLEMIDKLTTREIEQVELLKRIYDKL-OH were synthesized by Schafer-N (Copenhagen, Denmark).The purity of the peptides was above 80% as analysed by HPLC and mass spectrum.

### 2.4. Mouse Immunization

Female Balb/c mice were obtained from Taconic M&B (Ry, Denmark), 6–8 weeks old, and housed at the Panum Institute, University of Copenhagen. Mice were always allowed to acclimatize for at least one week before entering into experiments. All animal experiments were conducted in accordance with national guidelines. Mice were immunized with the recombinant fusion protein M2e-NSP4 or M2e peptide formulated with or w/o CAF-01 adjuvant (SSI, Copenhagen, Denmark), Freund’s complete (FCA) plus incomplete adjuvant (FIA) (Sigma-Aldrich) or Alhydrogel adjuvant (Brenntag Biosector A/S Fredrikssund, Denmark). Mice were immunized subcutaneously (s.c.) on days 0, 21, and 42. In some experiments, the immunization on day 42 was not performed. Blood samples were collected from the submandibular vein two weeks after each vaccination. The blood was allowed to clot prior to centrifugation, where after serum was obtained.

### Synthetic M2e Peptide ELISA

Nunc Maxisorb™ 96-well plates were coated overnight at 4°C with 100 µL/well of 300 ng/mL M2e peptide in 50 mM bicarbonate buffer, pH 9.6. The plates were washed 1× with PBS/Tween (phosphate buffered saline (PBS) containing NaCl (0.35 M) and 0.1% Tween-20, pH 7.4), and blocked for 1 hour at room temperature (RT) with a 2.5% casein solution. For ELISA, all plates were first washed 2× with PBS/Tween. The test samples and reagents were diluted in dilution buffer (0.35 M NaCl, 75 µM BSA, and 0.075% Tween in PBS, pH 7.2) at 100 µL/well and incubated for 1 hour at RT. Bound mouse antibodies were detected with HRP-labelled goat anti-mouse IgG antibodies (Fab-specific) (Sigma-Aldrich), rat anti-mouse IgG1 antibodies (BD PharMingen, San Diego, CA), rat anti-mouse IgG2a antibodies (BD PharMingen, San Diego, CA), goat anti-mouse IgM antibodies (Sigma-Aldrich) and goat anti-mouse IgA antibodies (Sigma-Aldrich). The plates were washed 2× with PBS/Tween and once with citric acid buffer (0.35 M C_6_H_8_O_7_.H_2_O and 0.67 M Na_2_HPO_4_.(H2O)_2_ in MilliQwater, pH 5) and then incubated for 30 min at dark with 100 µL/well of substrate buffer (citric acid buffer containing 0.7 mg/mL 1,2-phenylenediamine dihydrochloride (Kementec Diagnostics, Taastrup, Denmark) and 0.4 µL/mL 30% H_2_O_2_). The reaction was stopped with 0.2 M H_2_SO_4_ at 100 µL/well and the plates were read on the Multiskan® FC microplate photometer from Thermo Scientific. The antibody titer was defined as the reciprocal of the highest dilution that yielded an OD490 nm value 4 times above the mean value of the negative control wells.

### HeLa-M2 ELISA

HeLa enzyme-linked immunosorbent assay (ELISA) plates were prepared as follows: HeLa cells transfected with M2-expressing plasmid (HeLa-M2) encoding a tetracycline response element (Tet) and HeLa cells with an empty plasmid (HeLa-C10) were obtained from Robert B. Couch [13]. The cells were grown to confluency in Nunc Maxisorb™ 96-well plates by seeding wells with 1×10^5^ HeLa cells in 150 µL of Iscove’s Modified Dulbecco’s medium (Invitrogen) supplemented with 2-mercaptoethanol at 0.05 mM, transferrin (Sigma-Aldrich) at 0.005 mg/mL, gentamicin (Riedel-de Haën, Seelze, Germany) at 0.05 mg/mL, and 10% fetal bovine serum (FBS) (Lonza, Copenhagen, Denmark). The media was further supplemented with 1 µg/mL doxycycline (Sigma-Aldrich) for induction of Tet. After two days of incubation at 37°C in air/CO_2_, the media was flicked out and the cells were fixed with 0.05% glutaraldehyde (Sigma-Aldrich) for 20 min at RT. The plates were washed 2× with PBS/Tween and blocked with dilution buffer for 1 hour at RT or stored at 4°C. The test samples were diluted 1∶200 in dilution buffer at 50 µL/well and incubated for 1 hour at RT. Bound mouse antibodies were detected with HRP-labelled goat anti-mouse IgG antibodies (Fab-specific) (Sigma-Aldrich), The plates were washed 2× with PBS/Tween and 1× with citric acid buffer (0.35 M C_6_H_8_O_7_.H_2_O and 0.67 M Na_2_HPO_4_.(H2O)_2_ in MilliQwater, pH 5) and then incubated for 30 min at dark with 100 µL/well with substrate buffer (citric acid buffer containing 0.7 mg/mL 1,2-phenylenediamine dihydrochloride (Kementec Diagnostics, Taastrup, Denmark) and 0.4 µL/mL 30% H_2_O_2_). The reaction was stopped with 0.2 M H_2_SO_4_ at 50 µL/well and the plates were read at A_490_ and A_750_ on the Multiskan® FC microplate photometer from Thermo Scientific and the difference in OD_490–750_ between the HeLa-M2 and the HeLa-C10 plates was recorded. The difference between OD_490–750_ for HeLa-M2 and HeLa-C10 was recorded as ΔOD.

### HeLa-M2 Flowcytometric Assay

HeLa-M2 cells and HeLa-C10 cells were maintained as stated above. After 2 days of incubation with 1 µg/mL doxycycline (Sigma-Aldrich), 5×10^4^ cells were added into wells of 96-well round bottom micro-titer plates (Sigma-Aldrich). The cells were washed twice with washing buffer (PBS, 1% BSA, 5% FCS). Serum samples were added diluted 1∶100 in washing buffer at 50 µL/well and incubated at 4°C for 30 min. The plates were washed twice in washing buffer and the cells were incubated with 50 µL/well of FITC-conjugated Goat anti-mouse IgG2a specific (Jackson ImmunoResearch), PE-conjugated Goat anti-mouse IgG specific (Jackson ImmunoResearch) and APC-conjugated Goat anti-mouse IgG1 specific antibodies in washing buffer at 4°C for 30 min. The cells were washed twice in washing buffer and once in PBS. The cells were resuspended in 100 µL PBS and were analysed on a FACSCalibur (BD Biosciences). Mouse sera positive for all three stains and the monoclonal antibody 14C2 (Fisher Scientific Pierce MA1-082, AH diagnostics, Aarhus, Denmark) specific for M2e were used for instrument settings. A minimum of 10000 live cell events were normally collected. Data were analysed using the FlowJo program, and the difference in mean fluorescence intensity (MFI) between HeLa-M2 and the HeLa-C10 cells incubated with the same serum was recorded as ΔMFI.

### Virus Challenge of Mice

For evaluation of clinical protection, mice immunized as described above, were anaesthetized by intraperitoneal (i.p) injection with 250–300 µL Avertin (25 mg/mL) and infected intranasally (i.n.) with approx. 3 LD_50_ (dose lethal to 50% of mice) of either A/equine/London/72 (H7N7) or A/Puerto Rico/34 (PR8, H1N1). Survival and weight was monitored for up to 3 weeks; mice suffering a weight loss greater than 25% of their starting weight were euthanized for humane reasons.

### Quantitative-Reverse-Transcriptase-Polymerase-Chain-Reaction (QRT-PCR)

Viral loads were determined on 100 ng purified total RNA by duplex QRT-PCR using the Brilliant QRT-PCR Master Mix (Stratagene). Mice challenged with a lethal dose of PR8 were sacrificed by cervical dislocation at day 3 post infection. Lungs were removed and snap-frozen in either liquid nitrogen or dry ice. The tissues were homogenized in 3.6 mL lysis buffer (Macherey-Nagel, Düren, Germany) using a polytron. The tissue lysates were centrifuged twice at 3500 RPM for 10 min., and the supernatants were transferred to new tubes in between and after the second centrifugation. The clear tissue lysates were stored at −80°C. RNA from the homogenized tissues was extracted according to the standard kit extraction protocol from Nucleospin RNA II kit (Macherey-Nagel, Düren, Germany). RNA samples were quantified at 260 nm and qualitatively analysed by the 260/280 nm ratio. The samples were stored at −80°C before further use.

The primers used were directed against the M gene (5′ AGATGAGTCTTCTAACCGAGGTCG 3′ and 5′ TGCAAAAACATCTTCAAGTCTCTG 3′) and the housekeeping gene murine glyceraldehyde-3-phosphate dehydrogenase (mGAPDH) (5′ CAATGTGTCCGTCGTGGA 3′ and 5′ GATGCCTGCTTCACCACC 3′). The following probes were used, M2: FAM-TCAGGCCCCCTCAAAGCCGA-BHQ-1, mGAPDH: HEX-CGCCTGGAGAAACCTGCCAAGTAT-BHQ-1. The primers and the probes were synthesized by TAG Copenhagen A/S. The primer and probe sequences for the M gene had been previously published by Spackman *et al* [27]. The cycling conditions were: 30 min at 50°C, 10 min at 95°C followed by 40 cycles at 95°C for 30 s, 1 min at 58°C and 30 s at 72°C. All samples were run in triplicates and a standard curve was included in each run as an efficiency control of the amplification. A paCCMV plasmid encoding the coding region of the M gene had been previously constructed in the laboratory and was used for absolute quantifications. The copy number was determined with an optical density read out. The obtained results were analysed using the Stratagene MxPro software.

### Statistical Analysis

Antibody titers, viral titers, and MFIs were compared using the two-tailed Mann-Whitney rank test. Survival curves are presented as Kaplan-Meier plots and the statistical significance of differences between groups was determined by the log-rank test with the program GraphPad Prism (version 5.0).

## Results

### Purification of the M2e-NSP4 Protein

A construct containing the consensus sequence of M2e [11] fused to the C-terminal of the NSP4 fragment was cloned and expressed in *E. coli*. The fusion partner we have chosen has an easily predictable coiled-coil sequence. Coiled-coil sequences are known to be specific with respect to the number of subunits, and both the full-length native NSP4 as well as the coiled-coil region we have used as fusion partner for M2e are known to form stable tetramers [22]. The cultures were grown for induction in Erlenmayer shake flasks and the protein was purified by Ni-NTA and MonoQ chromatography. The purity of the protein was analysed by SDS-PAGE. Under reducing conditions, bands with a molecular weight of approximately 7 kDa could be observed, representing the monomer of the M2e-NSP4 construct (Fig. 1A). The M2e-NSP4 fusion protein elutes slightly faster than the 29-kDa carbonic anhydrase on a Superdex 75 gel filtration column, which is expected for tetrameric M2e-NSP4 (data not shown), and the native PAGE gel demonstrates that the protein is present as a single specimen (Fig. 1B).

### Immunogenicity of M2e and M2e-NSP4

To compare the immunogenicity of the recombinant fusion protein to that of free peptide, mice were immunized with 10 µg of antigen on days 0, 21 and 42, and sera collected 2 weeks after each immunization were analyzed for M2e-specific IgG antibodies by ELISA (Fig. 2). As standard adjuvant we chose CAF01, which is a synthetic two-component liposome-based adjuvant comprising the quaternary ammonium dimethyl-dioctadecyl-ammonium (DDA) and the immune modulator trehalose 6,6'-dibehenate (TDB) and likely to be approved for human use in the near future [23]–[26]. M2e-NSP4 immunization induced higher antibody titers after both the second and third immunization compared to M2e immunization, though the difference was not statistically significant.

In the same experiment we examined the requirement for linking M2e to the NSP4 fragment, acquiring an oligomerized construct, in order to obtain the same potency as seen with M2e-NSP4 immunization. Mice were immunized with a mix of 10 µg M2e and 10 µg of the NSP4 fragment and serum samples taken 2 weeks after each immunization were analyzed by ELISA. It was evident that linkage significantly increased the immunogenicity to M2e (Fig. 2).

The immunogenicity of the M2e-NSP4 vaccine was further investigated by comparing immunization with 100 µg M2e to 50 µg of M2e-NSP4. Increasing the M2e peptide dose ten-fold led to a marked increase in the antibody titer, which now matched that in mice immunized with the linked construct (Figure S1). However, increasing the M2e-NSP4 dose five-fold did not have any significant effect, indicating that a dose of 10 µg per immunization is optimal with this antigen. In summary, the results show that M2e-NSP4 is more potent than M2e when formulated in CAF-01, inducing a high antibody titer already after two immunizations with 10 µg, and that a 5 times higher dose of M2e-NSP4 does not further increase the response.

**Figure 3:**
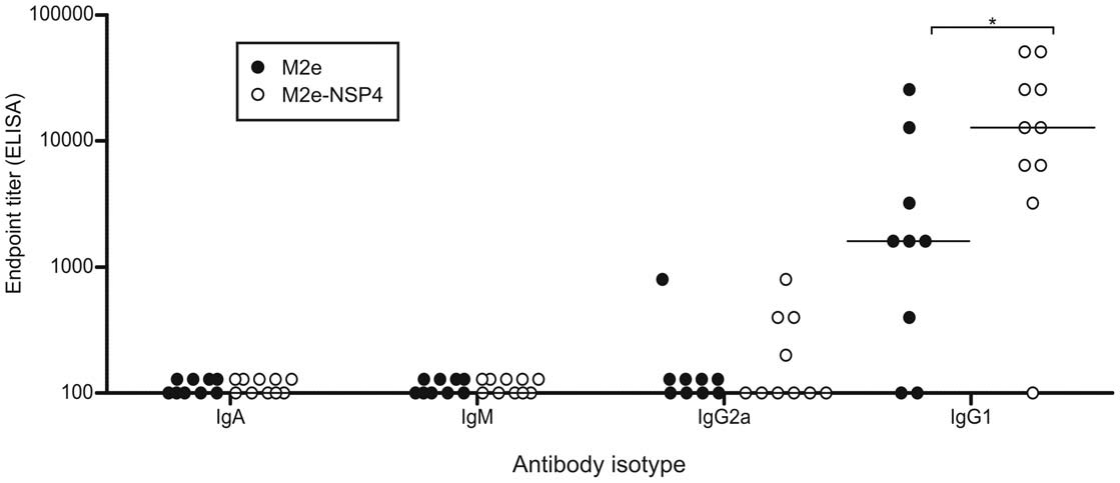
Isotype composition of M2e and M2e-NSP4 induced antibodies. Groups of Balb/c mice (n = 9–10) were immunized with 10 µg of M2e peptide or M2e-NSP4 formulated in CAF-01at days 0, 21, and 42. Serum samples obtained on day 56 after primary vaccination were analyzed in an ELISA for reactivity against M2e peptide. Results of individual sera are presented; group median titres are indicated by horizontal bars. *denotes a p-value <0.05.

Next, the isotypes of the M2e and M2e-NSP4 M2e induced antibodies after the third immunization were determined by ELISA. The main isotypes that were induced by both M2e and M2e-NSP4 immunization were IgG1, and to a lesser extent IgG2a (Fig. 3). M2e-NSP4 induced a significantly higher IgG1 antibody titer. No antibodies of the IgM or IgA isotypes were detected.

To test the efficacy of the CAF-01 adjuvant in comparison to other adjuvants commonly used in mouse experiments, the two vaccines were formulated with either CAF-01, AlOH, or FCA plus FIA (Figure S2). Formulating either vaccine with FCA plus FIA tended to increase the immunogenicity compared to CAF-01 formulation, while formulating the vaccines with AlOH did not. Notably, irrespective of adjuvant formulation, M2e-NSP4 was superior in immunogenicity to M2e.

**Figure 4:**
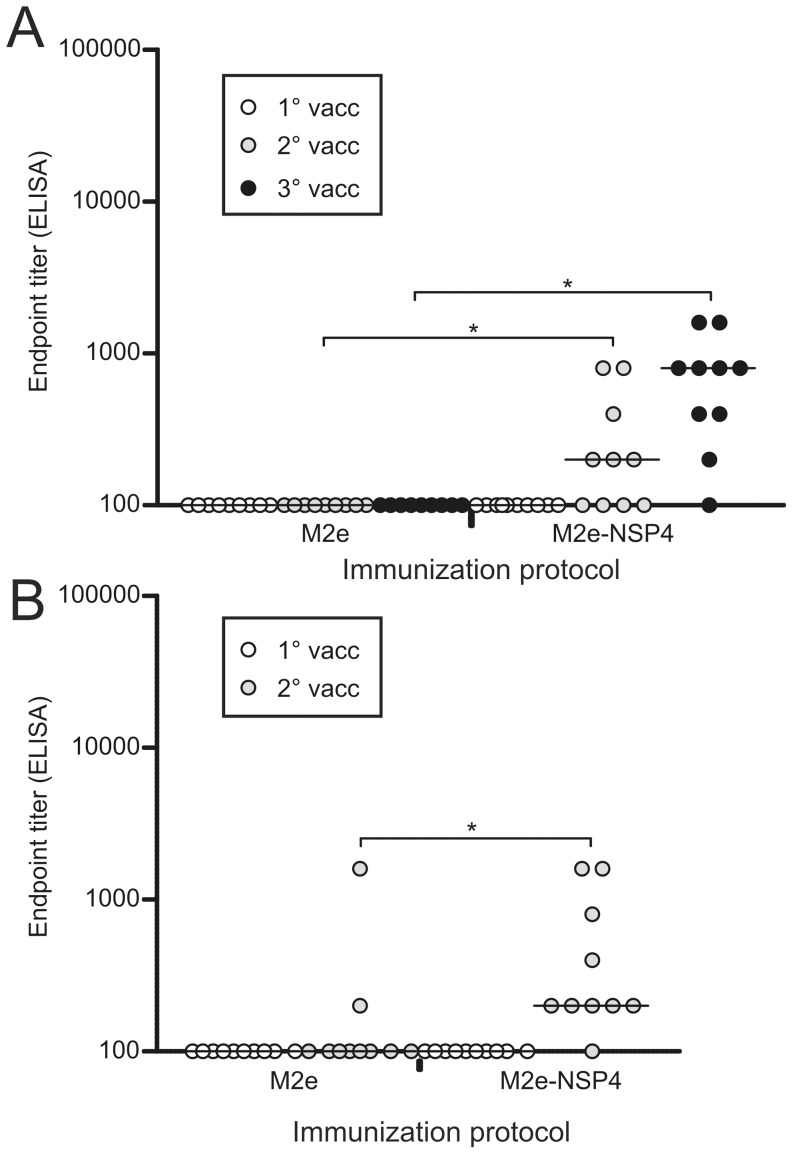
Comparison of immunogenicity of M2e-NS4 vs. M2e following truncation of the immunization regime. A) Groups of Balb/c mice (n = 9–10) were immunized with 1 µg of M2e peptide or M2e-NSP4 formulated in CAF-01at days 0, 21, and 42. Serum samples were obtained 14 days after each vaccination, i.e. at day 14 (1°vacc), 35 (2°vacc) and 56 (3°vacc) after primary vaccination, and analyzed in an ELISA for reactivity against M2e peptide. Results of individual sera are presented; group median titres are indicated by horizontal bars. *denotes a p-value <0.05. B) Groups of Balb/c mice (n = 10) were immunized with 10 µg of M2e peptide and M2e-NSP4 formulated in CAF-01at days 0 and 21. Serum samples were obtained at day 14 (1°) and 35 (2°) after primary vaccination and analyzed in an ELISA for reactivity against M2e peptide. Results of individual sera are presented; group median titres are indicated by horizontal bars. *denotes a p-value <0.05.

In the case of unpredicted influenza outbreaks, it might be of importance to spare on the available antigen. It was therefore investigated if antigen sparing by either lowering the antigen dose to 1 µg while preserving the immunization schedule or limiting the number of doses to two while maintaining the antigen dose at 10 µg would affect the potency of either vaccine. As can be seen in fig. 4, the M2e-NSP4 vaccine was superior in both cases, inducing significantly higher antibody titers both after two and three immunizations with 1 µg and after two immunizations with 10 µg compared to the M2e vaccine.

### Recognition of Native M2 in HeLa-M2 ELISA Assay

**Figure 5:**
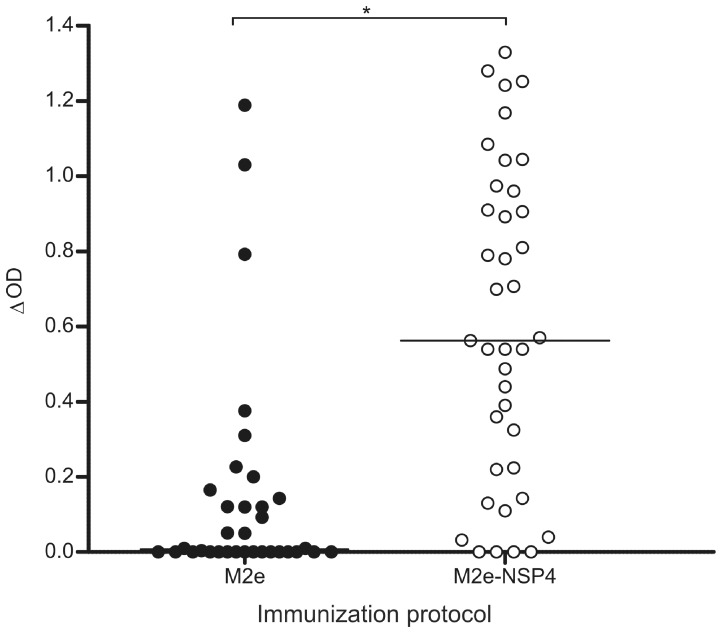
Reactivity of sera from M2e and M2e-NSP4 immunized mice against HeLa cells expressing M2 protein. Balb/c mice (n = 8–10/group/experiment) were immunized with 10 µg M2e peptide or M2e-NSP4 formulated in CAF-01 at days 0, 21, and 42. Serum samples harvested on day 56 after primary vaccination were analyzed in a cellular ELISA for reactivity to M2 protein as expressed on HeLa cells. Differences (Δ) in OD_490–750_ readings with M2 expressing and non-expressing control cells is depicted. Sera from naïve mice showed no preferential binding to M2-expressing cells (ΔOD = ∼0) and are not presented. Results of individual sera from 4 independent experiments are presented; group median titres are indicated by horizontal bars. *denotes a p-value <0.05.

The results presented above showed that antibodies induced by M2e as well as M2e-NSP4 immunization could react with synthetic consensus M2e peptide. It was next investigated whether the induced antibodies would also recognize the presumably native M2 protein, as expressed on transfected HeLa cells (HeLa-M2 cells). HeLa-M2 cells and HeLa-C10 cells (for evaluation of non-specific binding) were grown in 96-well plates in media supplemented with doxycycline for two days. The cells were then fixed and blocked, followed by incubation with individual serum samples (diluted 1∶200) obtained two weeks after the third immunization. Figure 5 shows the compiled results of four separate experiments. Matching what was observed in the ELISA using synthetic M2e peptide, a larger proportion of the mice immunized with M2e-NSP4 could recognize the native M2 protein compared to M2e immunized mice. Furthermore, sera from M2e-NSP4 immunized mice reached significantly higher median ΔOD values.

### Flowcytometric Analysis of Native M2 Recognition

**Figure 6:**
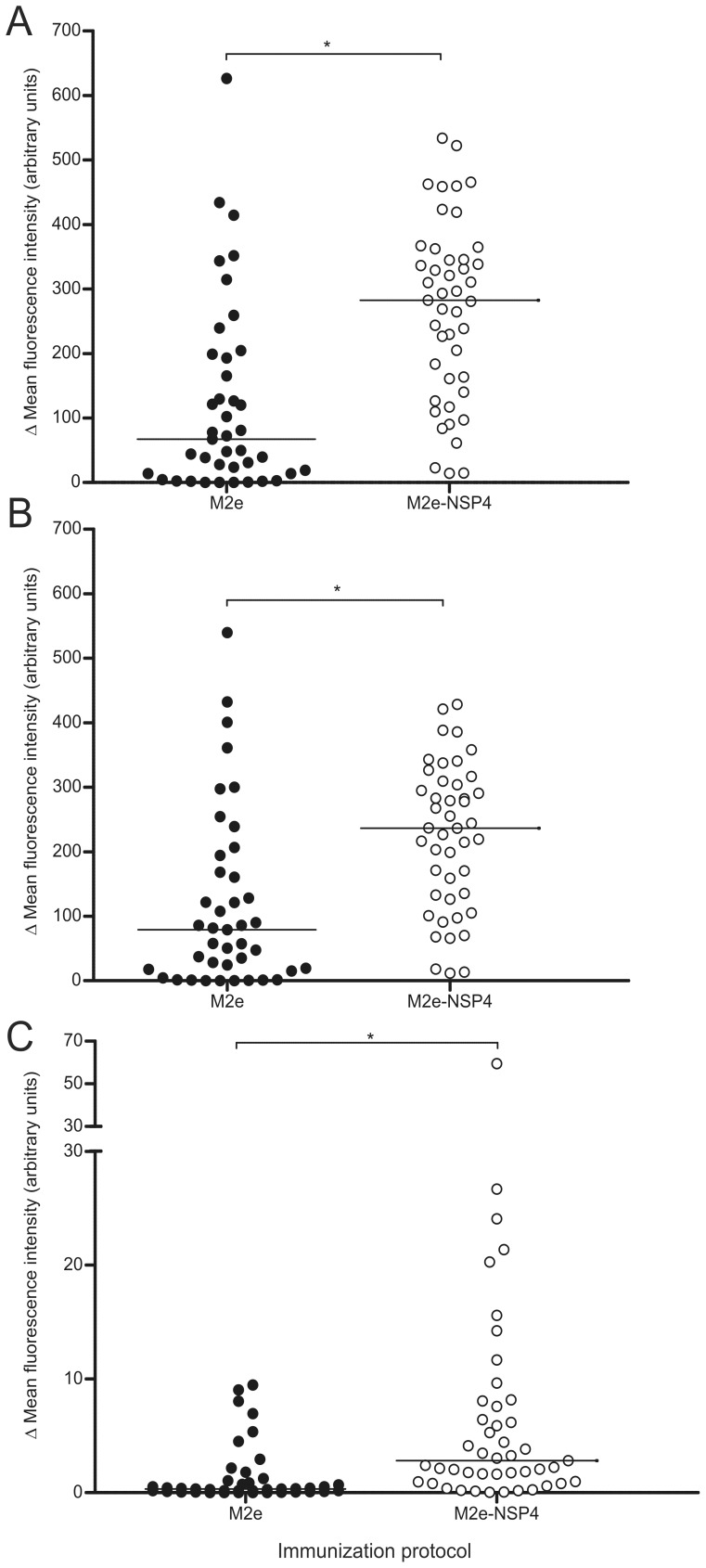
Reactivity of sera from M2e and M2e-NSP4 immunized mice against HeLa cells expressing M2 protein. Balb/c mice (n = 8–10/group/experiment) were immunized with 10 µg M2e peptide or M2e-NSP4 formulated in CAF-01 at days 0, 21, and 42. The isotype composition of M2-specific antibodies present in sera harvested on day 56 after primary vaccination was analyzed using flow cytometric analysis,differences in mean fluorescence intensitybetween M2-expressing and non-expressing HeLa cells is depicted: (A) total IgG, (B) IgG1, and (C) IgG2a. Results of individual sera from 4 independent experiments are presented; group median titres are indicated by horizontal bars. *denotes a p-value <0.05.

To confirm the above results and to investigate the isotype composition of the antibodies recognizing cell-expressed M2 protein, a flow cytometric analysis was performed using individual serum samples from the same four experiments analyzed above. HeLa-M2 and HeLa-C10 cells were treated as previously mentioned in the HeLa-M2 ELISA, and 5×10^4^ cells/well were added into 96-well plates. Serum samples (diluted 1∶100) were added next, followed by staining with isotype specific immunoconjugates. As expected, a significantly higher total IgG ΔMFI was observed for the M2e-NSP4 immunized mice (Fig. 6A, for representative histograms see Figure S3). The predominant M2-specific antibody isotype that was detected was IgG1 in both vaccination groups (Fig. 6B). Several serum samples from both M2e and M2e-NSP4 immunized mice were also found to contain M2-specific antibodies of the IgG2a isotype, although with a significantly higher median ΔMFI for M2e-NSP4 immunized mice (Fig. 6C). These results together with the results presented above indicate that the M2e-NSP4 vaccine is superior to M2e peptide, inducing significantly more antibodies with specificity for native M2 protein.

### Efficacy of M2e-NSP4 against Lethal Influenza Challenge

The ability of the vaccine to protect against a lethal influenza virus challenge was first investigated in mice immunized s.c. at day 0, 21, and 42 with 10 µg M2e-NSP4 with and without CAF-01, or with 1, or 0.1 µg M2e-NSP4 in CAF-01. Mice were challenged on day 81 by i.n. administration of a lethal dose of influenza A PR8 and weight and survival was monitored for three weeks (Figure S4). When formulated with CAF-01 the linked vaccine afforded significant protection compared to control mice. Interestingly, decreasing the dose of antigen ten-fold to 1 µg did not reduce the efficacy of the vaccine.

**Figure 7:**
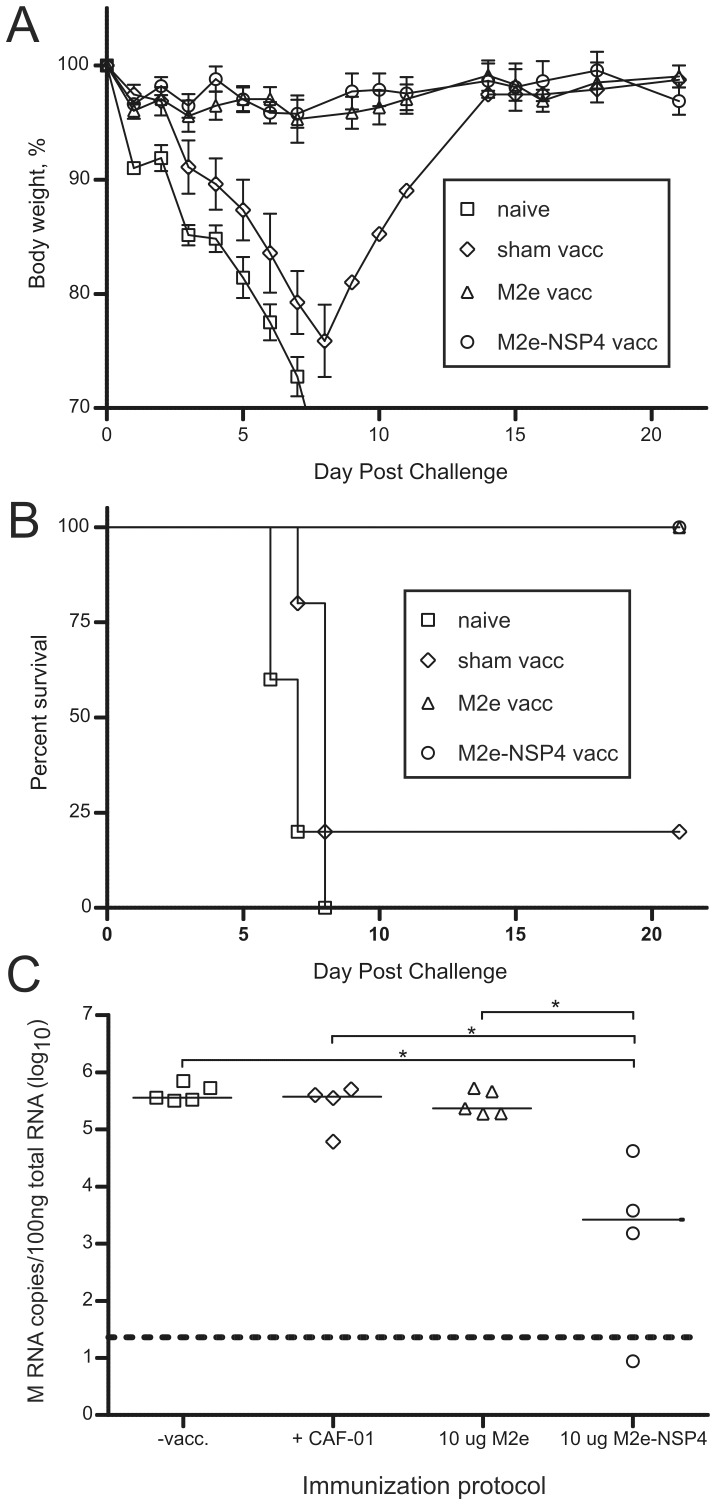
Comparison of the protection afforded by vaccination with M2e and M2e-NSP4 against challenge with an influenza virus closely related to the vaccine. Balb/c mice (n = 9–10/group) were immunized three times with 3 weeks interval with either adjuvant alone (CAF-01), 10 µg of M2e or 10 µg of M2e-NSP4 formulated in CAF-01. Around 4 weeks after last immunization the mice were challenged with 3 LD50 of influenza A virus PR8, differing by one amino acid from the M2e consensus sequence. The lungs from 4–5 mice from each group were removed for estimation of viral load 3 days after infection and analyzed by quantitative PCR as described in Materials and Methods (C). The remaining mice were monitored daily for survival (B) and loss of body weight (A). The survival curves are presented as Kaplan-Meier plots. *denotes a p-value <0.05. Data are representative of two experiments.

To evaluate the efficacy of M2e-NSP4 compared to M2e, groups of 9–10 mice were immunized three times with either 10 µg M2e or M2e-NSP4 s.c. and challenged with PR8 at day 69. At 3 d.p.i., lungs were removed from 4–5 mice from each group and from control mice for determination of lung viral titer with QRT-PCR. The remaining mice were observed for weight changes and survival. While no differences between M2e and M2e-NSP4 immunized mice could be observed with regard to weight change and survival (Fig. 7A and B), lung viral loads at 3 d.p.i. were found to be significantly lower in M2e-NSP4 immunized mice (Fig. 7C). This suggest that while the protection induced by immunization with M2e is sufficient to protect mice from severe weight loss and death, immunization with M2e-NSP4 seems to provide protection at an earlier stage causing also an early reduction in viral load in the lungs.

**Figure 8:**
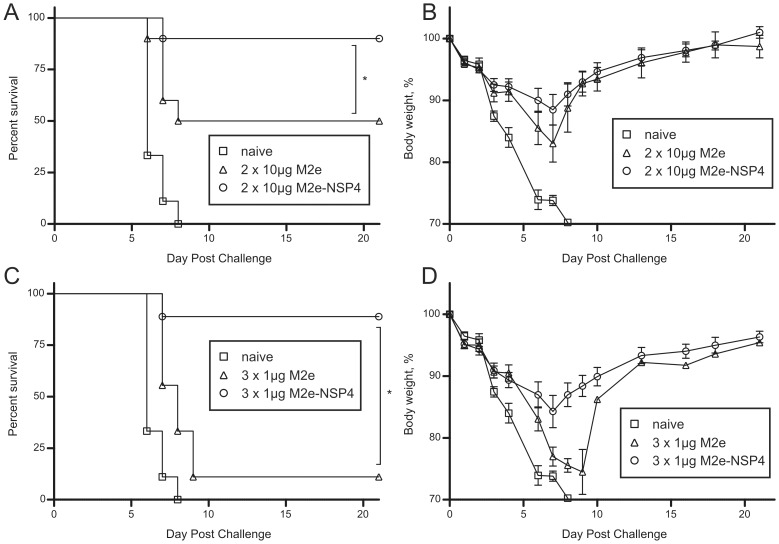
Impact of dose reduction on the protection afforded by M2e and M2e-NSP4 vaccination against challenge with a closely related influenza A virus. Balb/c mice (n = 9–10/group) were immunized either twice with 3 weeks intervals with 10 µg M2e or 10 µg M2e-NSP4 in CAF-01(A and B), or three times with 3 weeks intervals with 1 µg M2e or 1 µg M2e-NSP4 in CAF-01(C and D). Control mice were left untreated. Around 4–5 weeks after the last immunization all the mice were challenged with 3 LD50 of influenza virus PR8 and then monitored daily with regard to survival (A and C) and loss of body weight (B and D). *denotes a p-value <0.05.

Based on the encouraging results regarding analysis of viral load in the lungs, we decided to truncate the immunization regimen to investigate if this would lead to a difference also in survival rates. Consequently, 2 separate experimental set-ups were evaluated next. In the first, mice were immunized with the same dose as previously described, but challenged i.n. with a lethal dose of PR8 already after the second immunization. In the second set-up, the full sequence of the immunization protocol was applied, but the antigen doses used for each vaccination were reduced by a factor of 10 - from 10 to 1 µg. As can be seen in fig. 8, only half the challenged mice survived a lethal challenge if the mice were vaccinated only twice with the M2e peptide, whereas all but one animal survived if the mice had been vaccinated twice with the standard dose of M2e-NSP4 (fig. 8A and B). Even more clearly, if the vaccine dose was reduced to one tenth of the original, nearly all peptide vaccinated mice succumbed to an otherwise lethal infection, while all but one M2e-NSP4 vaccinated mouse resisted the challenge (fig. 8C and D). Taken together, these results strongly support the idea that immunization with M2e in a tetramerized form induces substantially better clinical protection than immunization with the monomeric peptide.

### Cross-reactivity of Anti-M2 Antibodies

**Figure 9:**
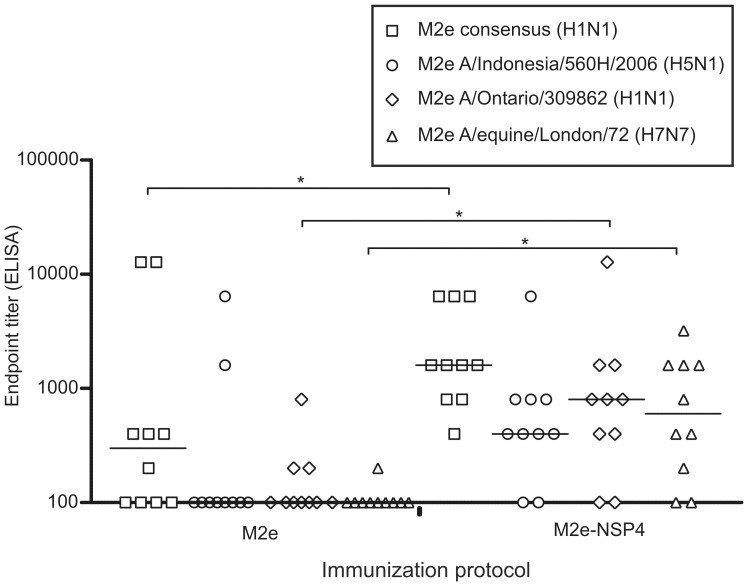
Cross-reactivity of M2e-specific antibodies generated in M2e and M2e-NSP4 vaccinated mice. Groups of Balb/c mice (n = 9–10) were immunized with either 10 µg M2e or M2e-NSP4 formulated in CAF-01at days 0, 21, and 42. Serum samples obtained at day 56 after primary vaccination were analyzed in an ELISA for reactivity against various M2e peptides: M2e consensus sequence, M2e A/Indonesia/560H/2006 (H5N1), M2e A/Ontario/309862 (H1N1), M2e A/equine/London/72 (H7N7). Results of individual sera are presented; group median titres are indicated by horizontal bars. *denotes a p-value <0.05. Data are representative of at least three experiments.

To explore whether immunization with M2e-NSP4 had the potential to afford a broader protection against virus strains not expressing the M2e consensus sequence, we assessed the cross-reactivity of M2e-NSP4 and M2e induced antibodies in an ELISA assay with different variants of synthetic M2e peptides. Individual serum samples from the third immunization of a representative experiment were tested for their reactivity to consensus M2e, swine-origin H1N1 M2e, avian-origin H5N1 M2e, and equine H7N7 M2e. As can be seen in Table 1, the swine and avian derived sequences differ by 4 and 3 amino acids, respectively, from the consensus sequence, while the equine sequence differs by 5 amino acids. As can be seen in fig. 9, antibodies induced by M2e-NSP4 immunization were significantly better at cross-reacting with the swine and equine peptides but not the avian peptide than were antibodies from M2e immunized mice. Taken together, these results imply that M2e-NSP4 induces more broadly reactive antibodies and the fusion vaccine might therefore potentially be effective against a wider range of influenza A virus strains.

**Table 1:** Amino acid sequences from M2e.

Strain	Subtype	Origin	Amino Acid Sequence*
Consensus	H1N1	Human	SLLTEVETPIRNEWGCRCNDSSD
A/Puerto Rico/34	H1N1	Human	SLLTEVETPIRNEWGCRCN**G**SSD
A/equine/London/73	H7N7	Equine	SLLTEVETP**TKS**EW**E**CRCN**V**SSD
A/Ontario/309862/2009	H1N1	Swine	SLLTEVETP**T**R**S**EW**E**CRC**S**DSSD
A/Indonesia/560H/2006	H5N1	Avian	SLLTEVETP**T**RNEW**E**CRC**S**DSSD

* Sequences are compared to consensus, and mutations are indicated in bold.

**Figure 10:**
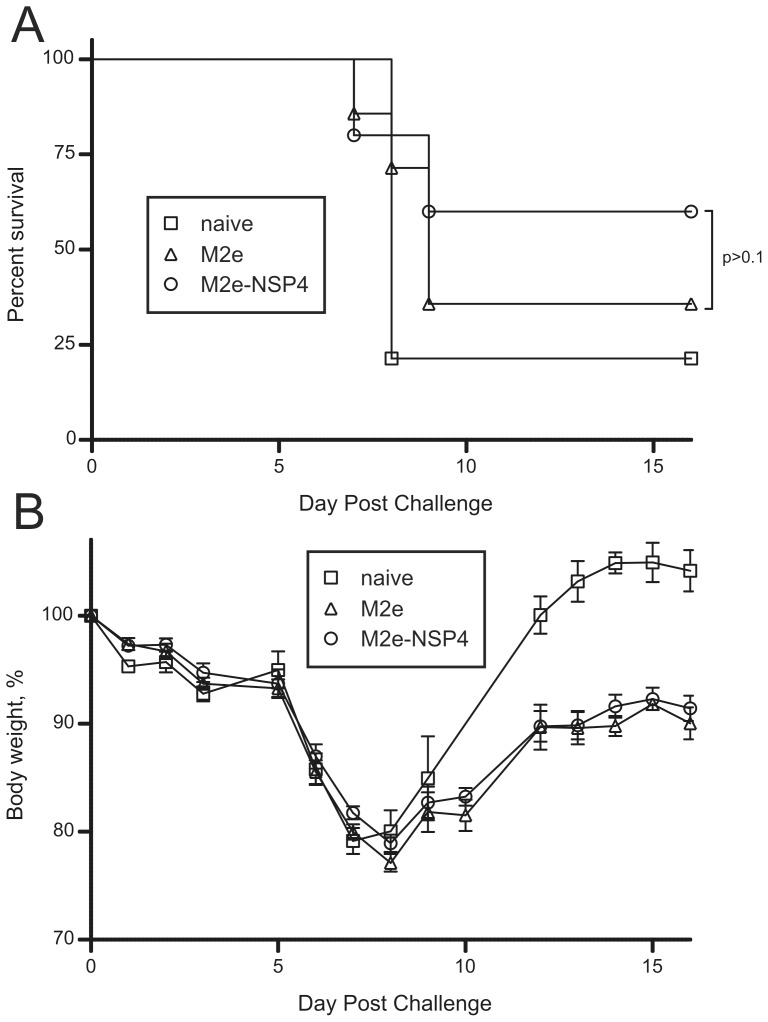
Comparison of M2e and M2e-NSP4 induced protection against challenge with a distantly related influenza A virus. Balb/c mice (n = 5–10/group//experiment) were immunized three times with 3 weeks interval with either 10 µg M2e or 10 µg M2e-NSP4 in CAF-01. Control mice were left untreated. Around 4 weeks after the last immunization the mice were infected with 3 LD50 of influenza virus H7N7, differing by 5 amino acids from the M2e consensus sequence. Mice were monitored for survival (A) and loss of body weight (B) daily.

Finally, to determine if the M2e-NSP4 vaccine could protect against infection with a heterosubtypic influenza virus, two groups of mice were immunized three times s.c. with either 10 µg of M2e or 10 µg of M2e-NSP4, and in two separate experiments these mice were challenged with a lethal dose of H7N7, differing from the M2e consensus sequence by 5 amino acids. Body weight changes (data not shown) and survival were monitored during 16 days post challenge (Fig.10). It was observed that vaccination with M2e-NSP4 tended to provide better protection against a heterosubtypic influenza infection although the difference was not statistically significant with the numbers of mice investigated here.

## Discussion

The trivalent influenza vaccine that includes the viral surface proteins HA and NA, is the primary vaccine in use against seasonal influenza epidemics. This vaccine is quite efficient at inducing neutralizing antibodies, which can then protect against the viral strains circulating during a specific season. However, a vaccine that would not have to be updated every season would have major economical and health benefits, particularly in developing countries where annual vaccination is not easily applicable. Recent studies have led to the discovery and targeting of highly conserved regions in the HA protein showing significant protective potential [28]–[30]. However, even if these candidates seem promising, they still face the issue of only inducing group-specific protection [31]. On the other hand, having a universal vaccine solely based on the M2 protein is not without its problems either, since there still does not exist a standardized protocol for the ex vivo validation of the protection induced by M2 based vaccines. This very substantially increases the cost involved in introducing an M2–based vaccine on the market. However, thus far, groups with promising M2 targeting candidates have steered clear of this dilemma by including HA antigen in their vaccines [19], [32].

In this study we characterized and analyzed the potential of a M2 vaccine construct intended to mimick the native structure of M2e. Thus, consensus sequence M2e was fused to the NSP4_98–135_ fragment of rotavirus, giving a fusion protein (M2e-NSP4) in which the tetramerizing NSP4 fragment would structurally replace the transmembrane section of the native M2 protein. This would allow for four M2e fragments to aggregate and thus mimic the native viral M2e conformation. When compared to M2e peptide immunization in side-by-side analysis and with both vaccines formulated in CAF-01 adjuvant, it was clearly revealed that M2e-NSP4 was more immunogenic and could be used at lower doses than the M2e peptide without loosing protective efficacy against closely related influenza A virus challenge. At higher antigen doses, both vaccines induced similar M2e peptide specific antibody responses, though the early reduction of the lung viral titers in PR8-challenged M2e-NSP4 immunized mice compared to M2e peptide vaccinated mice could indicate that the more efficient recognition of native M2 protein in the former mice might be associated with better clinical protection.

As antibodies recognizing conformational epitopes might prove to be more relevant in relation to in vivo protection, two assays were used to analyze the reactivity of the induced antibodies to native M2 protein as expressed on HeLa-M2 cells. It has previously been demonstrated that assays using native M2 protein as a basis for the assessment of vaccine-induced antibodies provide more relevant information than M2e-peptide based assays [33]. The superiority of M2e-NSP4 that was implied in the M2e-peptide ELISA assay became more evident in the two assays based on HeLa cells expressing native M2 protein. In both the HeLa-M2 ELISA and the HeLa-M2 FACS assay, M2e-NSP4 was significantly better at inducing M2 specific antibodies.

For a universal vaccine to be of any real relevance as an add-on vaccine to the normal seasonal vaccine and/or as an interim pandemic influenza vaccine it needs to be cross-protective. Several attempts have been made to induce cross-reactive and therefore cross-protective antibodies based on the highly conserved M2e sequence. Although the M2e sequence is almost invariant within human influenza A viruses there are several aa differences between the human consensus sequence and avian sequences [34]. In a study by Fan *et al.* the cross-reactivity of antibodies induced by M2e-carrier constructs based on M2e sequences from the consensus or the PR8 sequence were tested against several M2e-peptides, and no cross-reactivity was observed with avian M2e-peptides that differed by 3–4 aa from the consensus sequence [8]. The only study that has been successful in demonstrating protection against an influenza A virus subtype encoding an M2e differing substantially from the consensus is that of Tompkins *et al*. [18]. Their challenge virus differed by five aa and vaccination protected 80% of the mice against lethal challenge. The antibodies induced by M2e-NSP4 vaccination could cross-react with M2e-peptides differing by five aa from the vaccine M2e sequence (Fig. 9). Upon challenge with H7N7 virus differing by the same number of aa, the protection of M2e-NSP4 immunized mice was slightly better than that of M2e peptide immunized mice (Fig. 10); however, only 60% of the M2e-NSP4 mice were protected. In this context it should be noted that compared to the challenge virus used by Tompkins *et al.*, which was of the H1N1 subtype, H7N7 is considered to be a highly pathogenic subtype [35], and the neurotropism of H7N7 may be a complicating factor making this model less optimal when trying to evaluate antibody based protection.

To conclude, in this study we show that M2e-NSP4 is superior to M2e-peptide immunization in inducing antibodies against the native M2 protein. Additionally, the M2e-NSP4 construct is very cheap and easy to produce in large quantities. In the case where both vaccines were equally good at providing full protection against lethal challenge with a virus differing from the vaccine sequence by one aa, only M2e-NSP4 immunized mice presented with a significantly reduced early virus load in the lungs. Moreover, following a reduction in either the number of vaccine doses or the amount of antigen used for each immunization, M2e-NSP4 was clearly superior at inducing protective immunity. Finally, M2e-NSP4 immunization induced antibodies that were significantly more cross-reactive to non-consensus M2e peptides and tended to afford better protection against a virus differing by five aa from the M2e consensus sequence.

## Supporting Information

Figure S1 **Dose/response comparison of M2e and M2e-NSP4 immunization.** Groups of Balb/c mice (n = 9–10) were immunized at days 0, 21, and 42 with 10 or 100 µg of M2e peptide or 10 and 50 µg of M2e-NSP4, both formulated in CAF-01. Serum samples were obtained 14 days after each vaccination, i.e. at days 14 (1°vacc), 35 (2°vacc), and 56 (3°vacc) after primary vaccination and analyzed in an ELISA for reactivity against M2e peptide. Results of individual sera are presented; group median titres are indicated by horizontal bars. *denotes a p-value <0.05. The results are representative of two experiments.
(TIF)

Figure S2 **Comparison of adjuvants.** Groups of Balb/c mice (n = 5) were immunized with 10 µg M2e peptide or M2e-NSP4 formulated in CAF-01, FIA + FCA, or AlOH at day 0, 21, and 42. Serum samples obtained on day 56 after primary vaccination were analyzed in ELISA for reactivity against M2e peptide. Results of individual sera are presented; group median titres are indicated by horizontal bars. *denotes a p-value <0.05. The data shown are representative of two experiments.
(TIF)

Figure S3 **Reactivity of sera from M2e and M2e-NSP4 immunized mice against HeLa cells expressing M2 protein.** Balb/c mice were immunized with 10 µg M2e peptide or M2e-NSP4 formulated in CAF-01 at days 0, 21, and 42. Binding of antibodies to HeLa cells expressing M2e (HeLa-M2) and non-expressing control cells (HeLa-C10) was analyzed by flowcytometry using sera harvested on day 56 after primary vaccination, representative histograms are presented. Binding of the M2e-specific monoclonal antibody 14C2 served as a positive control.
(TIF)

Figure S4 **Evaluation of dose of M2e-NSP4 vaccine required for in vivo protection.** Balb/c mice (n = 4–5) were immunized three times with 3 weeks interval with either 10 µg M2e-NSP4 with or w/o CAF-01, or 1 or 0.1 µg M2e-NSP4 in CAF-01. Control mice were left untreated. Around 6 weeks after the last immunization the mice were infected with 3 LD50 of influenza virus PR8. The mice were monitored daily with regard to survival (A) and loss of body weight (B).
(TIF)
