# Supplementary figures and images for: Increased Immunogenicity and Protective Efficacy of Influenza M2e Fused to a Tetramerizing Protein
Source: PLoS One. 2012 Oct 1;7(10):e46395. doi: 10.1371/journal.pone.0046395 (PMC3462204; doi:10.1371/journal.pone.0046395)

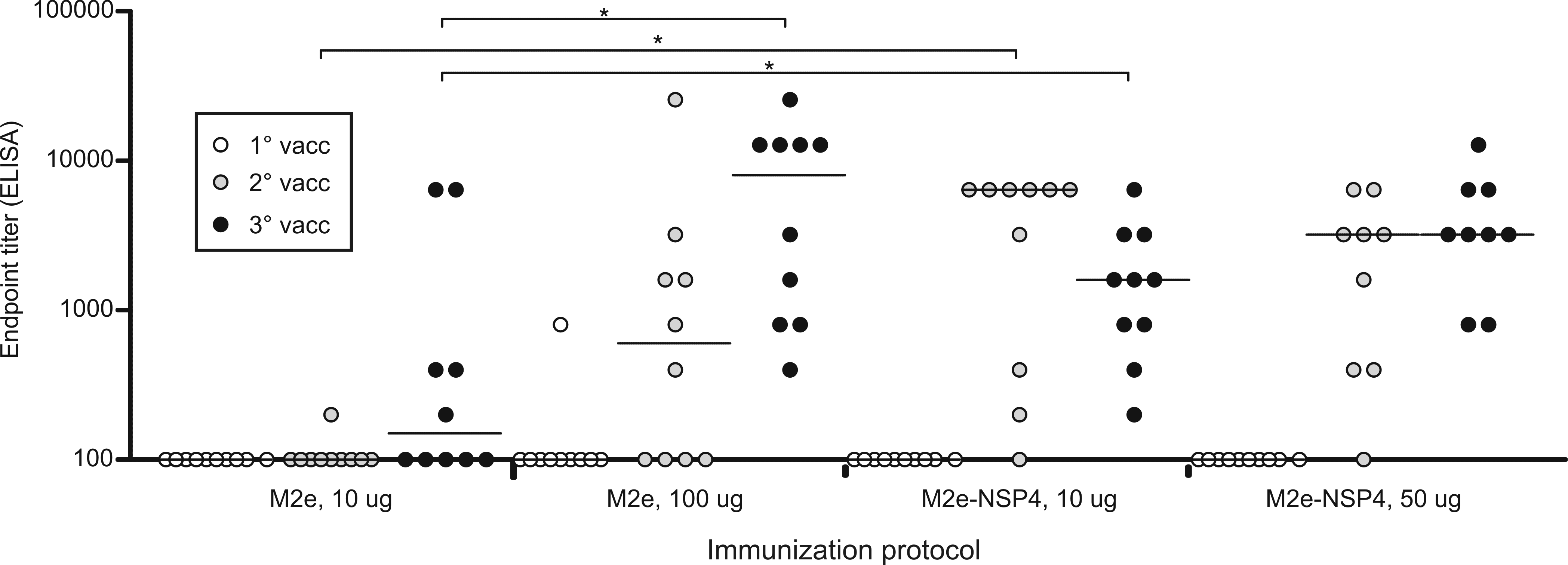

Supplement: Figure S1 — Dose/response comparison of M2e and M2e-NSP4 immunization. Groups of Balb/c mice (n = 9–10) were immunized at days 0, 21, and 42 with 10 or 100 µg of M2e peptide or 10 and 50 µg of M2e-NSP4, both formulated in CAF-01. Serum samples were obtained 14 days after each vaccination, i.e. at days 14 (1°vacc), 35 (2°vacc), and 56 (3°vacc) after primary vaccination and analyzed in an ELISA for reactivity against M2e peptide. Results of individual sera are presented; group median titres are indicated by horizontal bars. *denotes a p-value <0.05. The results are representative of two experiments. (TIF) [file pone.0046395.s001.tif]

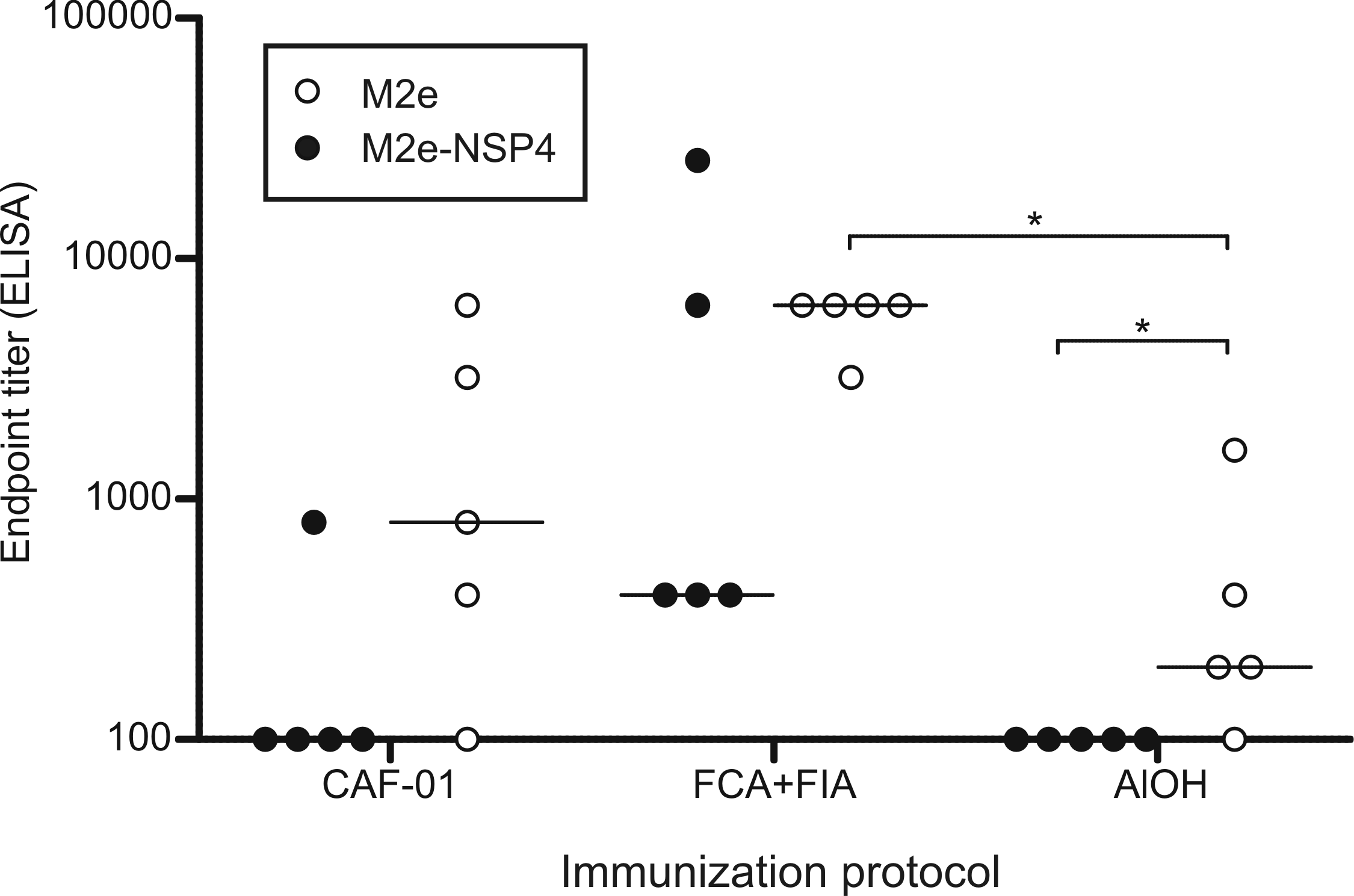

Supplement: Figure S2 — Comparison of adjuvants. Groups of Balb/c mice (n = 5) were immunized with 10 µg M2e peptide or M2e-NSP4 formulated in CAF-01, FIA + FCA, or AlOH at day 0, 21, and 42. Serum samples obtained on day 56 after primary vaccination were analyzed in ELISA for reactivity against M2e peptide. Results of individual sera are presented; group median titres are indicated by horizontal bars. *denotes a p-value <0.05. The data shown are representative of two experiments. (TIF) [file pone.0046395.s002.tif]

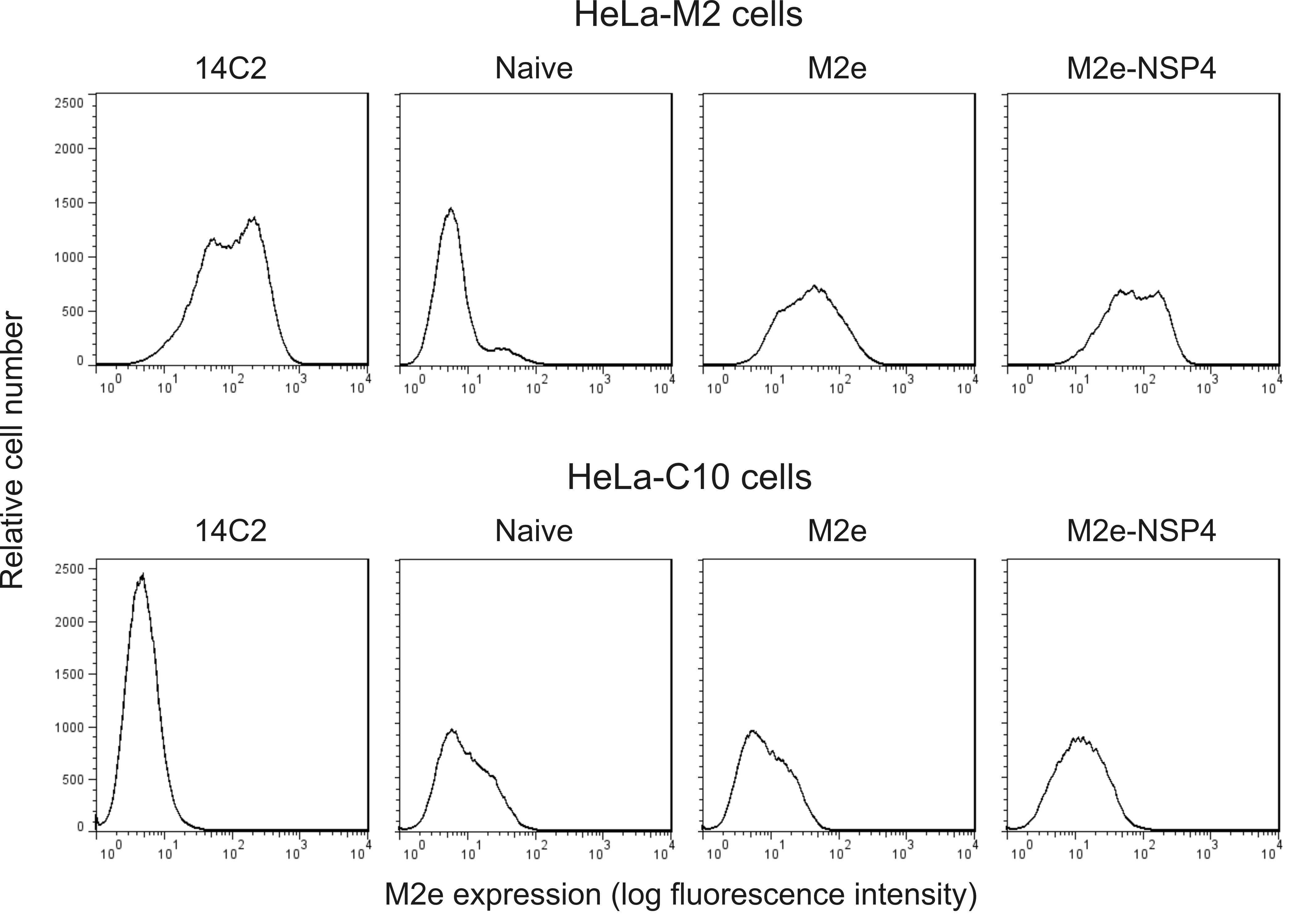

Supplement: Figure S3 — Reactivity of sera from M2e and M2e-NSP4 immunized mice against HeLa cells expressing M2 protein. Balb/c mice were immunized with 10 µg M2e peptide or M2e-NSP4 formulated in CAF-01 at days 0, 21, and 42. Binding of antibodies to HeLa cells expressing M2e (HeLa-M2) and non-expressing control cells (HeLa-C10) was analyzed by flowcytometry using sera harvested on day 56 after primary vaccination, representative histograms are presented. Binding of the M2e-specific monoclonal antibody 14C2 served as a positive control. (TIF) [file pone.0046395.s003.tif]

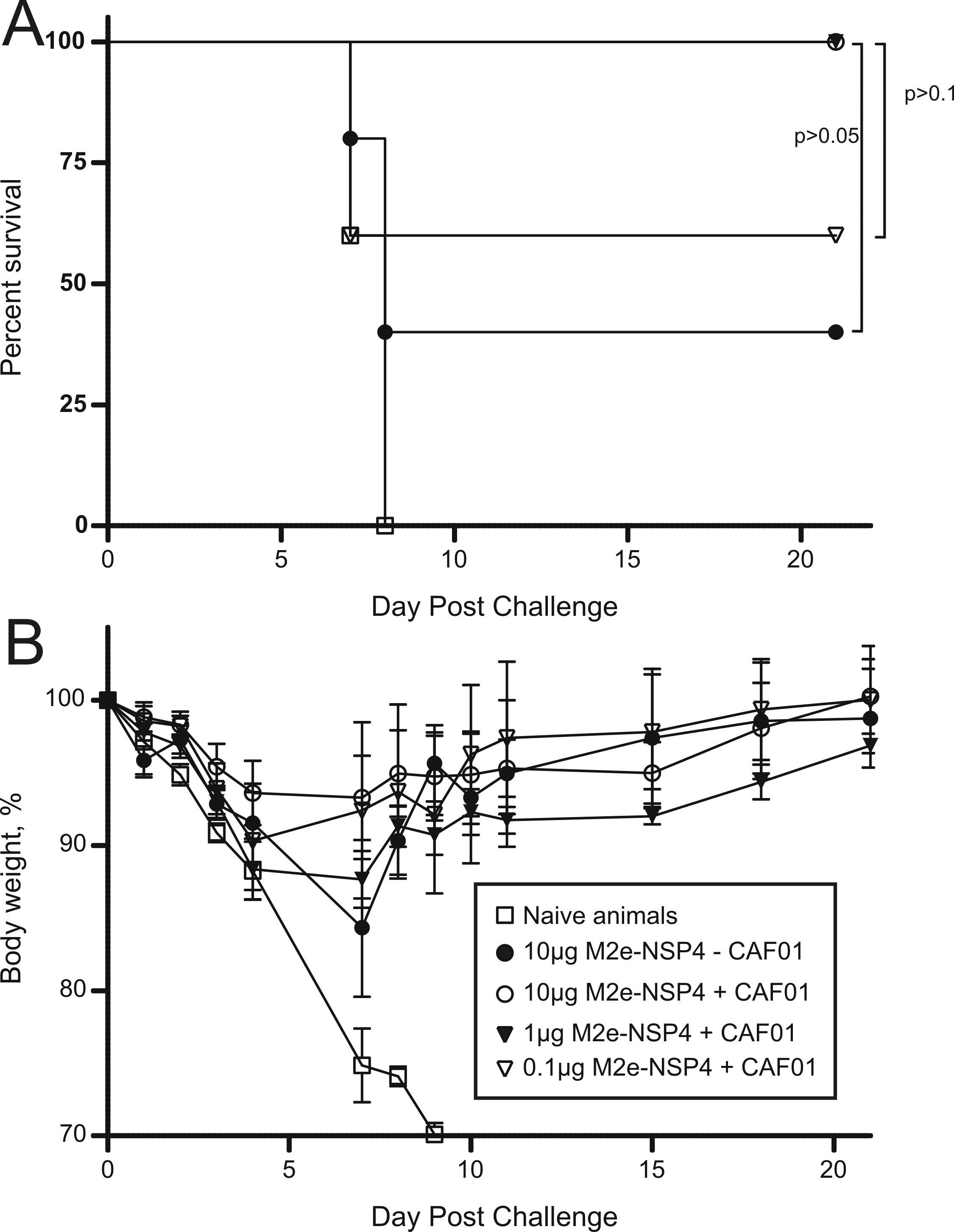

Supplement: Figure S4 — Evaluation of dose of M2e-NSP4 vaccine required for in vivo protection. Balb/c mice (n = 4–5) were immunized three times with 3 weeks interval with either 10 µg M2e-NSP4 with or w/o CAF-01, or 1 or 0.1 µg M2e-NSP4 in CAF-01. Control mice were left untreated. Around 6 weeks after the last immunization the mice were infected with 3 LD50 of influenza virus PR8. The mice were monitored daily with regard to survival (A) and loss of body weight (B). (TIF) [file pone.0046395.s004.tif]
